# Prevalence of Medical Conditions Potentially Amenable to Cellular Therapy among Families Privately Storing Umbilical Cord Blood

**DOI:** 10.1007/s10995-016-2110-1

**Published:** 2016-08-16

**Authors:** Peter Mazonson, Mark Kane, Kelin Colberg, Heather Harris, Heather Brown, Andrew Mohr, Alyssa Ziman, Chris Santas

**Affiliations:** 1 Mazonson & Santas, Inc., 15 Hillcrest Avenue, Larkspur, CA 94939 USA; 2 Scientific and Medical Affairs, CBR Systems, Inc., 1200 Bayhill Drive, 3rd Floor, San Bruno, CA 94066 USA; 3 Department of Pathology and Laboratory Medicine, David Geffen School of Medicine at UCLA, 10833 Le Conte Avenue, Los Angeles, CA 90095 USA

**Keywords:** Hematopoietic stem cells, Stem cell transplant, Regenerative medicine, Cord blood stem cell transplantation, Umbilical cord blood, Blood bank

## Abstract

*Introduction* Little is known about the prevalence of conditions potentially amenable to cellular therapy among families storing umbilical cord blood in private cord blood banks. *Methods* A cross-sectional study of families with at least one child who stored umbilical cord blood in the largest private cord blood bank in the United States was performed. Respondent families completed a questionnaire to determine whether children with stored cord blood or a first-degree relative had one or more of 16 conditions amenable primarily to allogeneic stem cell transplant (“transplant indications”) or 16 conditions under investigation for autologous stem cell infusion (“regenerative indications”), regardless of whether they received a transplant or infusion. *Results* 94,803 families responded, representing 33.3 % of those surveyed. Of respondent families, 16.01 % indicated at least one specified condition. 1.64 % reported at least one first-degree member with a transplant indication potentially treatable with an allogeneic stem cell transplant. The most common transplant indications reported among first-degree family members were Non-Hodgkin’s Lymphoma (0.33 %), Hodgkin’s Lymphoma (0.30 %), and Acute Lymphoblastic Leukemia (0.28 %). 4.23 % reported at least one child with a regenerative indication potentially treatable with an autologous stem cell infusion. The most common regenerative indications among children with stored umbilical cord blood were Autism/Autism Spectrum Disorder/Apraxia (1.93 %), Other Developmental Delay (1.36 %), and Congenital Heart Defect (0.87 %). *Discussion* Among families storing umbilical cord blood in private cord blood banks, conditions for which stem cell transplant or infusion may be indicated, or are under investigation, appear to be prevalent, especially for regenerative medicine indications.

**Electronic supplementary material:**

The online version of this article (doi:10.1007/s10995-016-2110-1) contains supplementary material, which is available to authorized users.

## Significance

The identification of umbilical cord blood (UCB) as a source of stem cells has made cord blood banking an important topic. Yet, among families with stored UCB, the prevalence of conditions either known to be treatable or under investigation for treatment with UCB is largely unknown. We believe this is the first study of disease prevalence in a private cord blood bank. Among families surveyed, 1.64 % reported at least one first-degree member with an indication potentially treatable with an allogeneic stem cell transplant, while 4.23 % reported at least one child with an indication under investigation for treatment with an autologous stem cell infusion.

## Introduction

Umbilical cord blood (UCB) is a rich source of hematopoietic stem cells. Since the first successful sibling cord blood transplant for Fanconi anemia in 1988, UCB has been used to treat a variety of life-threatening conditions, including hematologic malignancies, hemoglobinopathies, and metabolic and immune disorders (Gluckman et al. 2011). More recently, research has suggested that UCB has the potential to play a role in regenerative medicine applications where it may promote repair of organs and tissues outside of its hematopoietic lineage (Harris et al. 2007; Willert et al. 2008). In these applications, it is thought that UCB may repair damaged tissues either via cell differentiation and replacement or, more likely, through the release of anti-inflammatory and other factors that stimulate endogenous repair mechanisms (Willert et al. 2008; Hau et al. 2008; Neuhoff et al. 2007). As a result, new applications for UCB are now actively being investigated in the laboratory and in clinical trials. Currently, ten US- based clinical trials are being conducted in pediatric populations to investigate the regenerative medicine potential of UCB (Electronic Resource 1). A range of conditions are being studied including cerebral palsy, hearing loss, hypoplastic left heart syndrome, hypoxic-ischemic encephalopathy, pediatric stroke, type I diabetes, and autism (“US National Institutes,” 2015).

Traditionally, in transplant medicine, UCB from a healthy, human leukocyte antigen (HLA)-matched donor is used in an allogeneic transplant to repopulate the recipient’s bone marrow after a pre-conditioning regimen. In contrast, in the United States, the experimental use of UCB for regenerative medicine indications is often referred to as a stem cell “infusion” and almost always autologous. Therefore, although autologous transplants may be performed for traditional indications and allogeneic infusions may be performed for regenerative indications, for the purpose of this study, the term “transplant indications” refers exclusively to medical conditions for which UCB transplants are more likely to be done in an allogeneic fashion whereas, the term “regenerative indications” refers exclusively to medical conditions for which UCB infusions are more be likely to be done in an autologous fashion.

Until recently, UCB was considered medical waste (Forraz and McGuckin 2011; Badowski and Harris 2012). Soon after the first successful demonstration of UCB as a stem cell source, cord blood banking was established in the United States. As of 2013, it was conservatively estimated that at least 1.36 million cord blood units were banked in the United States (“Parent’s Guide to Cord Blood,” 2014). Families have the choice to either store cord blood in a private cord blood bank or to donate it to the public cord blood banking system. Private and public cord blood banks are designed to serve different needs. For a fee, private banks reserve the cord blood unit exclusively for use by the donor and immediate family members, while UCB donated to a public bank may be used by any patient in need, though rarely the actual donor. Private cord blood banking continues to grow. According to a 2009 estimate, private cord blood banks now store approximately 60 % of the more than half million UCB units processed each year worldwide (“World Stem Cell Summit,” 2009).

Despite growing participation, little information has been collected on families who choose to privately bank their children’s UCB. This study was carried out to better characterize this population and to determine the prevalence of diseases and conditions potentially amenable to UCB transplants or infusions among families at a large private cord blood bank.

## Methods

### Study Population and Design

We conducted a cross-sectional survey using an online, one-page Family Health Questionnaire (FHQ) of self-reported disease prevalence among all eligible families with at least one living child with stored UCB. These families were identified using a contact database maintained by the largest private cord blood bank in the United States. All families that elected to collect and store UCB from January 1994 to May 2014 were initially included. Families were ineligible to receive an FHQ if they did not have a valid e-mail address on file in the company database, had a “do-not-contact” request in place, had a deceased child with stored UCB, or had a deceased primary parent contact. Additionally, families with only adopted or surrogate births were not included due to the FHQ’s focus on biological relatives. These exclusions accounted for approximately 17 % of the active storage population.

### Survey Instrument

The FHQ was designed to determine whether a child with stored UCB, or a first-degree relative of that child, currently had one or more listed diseases or conditions. This survey captured self-reported information from the child’s primary caregiver using check boxes for 16 transplant indications and for 16 regenerative indications. The transplant indications were selected based on documented efficacy of UCB transplants for these indications, while the regenerative indications were selected based on clinical trials and other case series designed to explore the efficacy of UCB for those indications. Information was collected regardless of whether families had already received or planned to receive a transplant or infusion. Instructions as well as access to the FHQ were sent to eligible families via e-mail. The questionnaire was sent in two main waves in October 2013 and May 2014. After initial contact, non-responding families were reminded via e-mail at least two more times to complete the FHQ. These data were collected as a preliminary activity for an IRB-approved, longitudinal study (E&I Review Board).

### Statistical Analysis

Available demographic information was extracted from cord blood bank enrollment forms and used to compare responders versus non-responders. Potential non-response bias based on respondent age, age of children with stored cord blood, and number of stored cord blood units per family was assessed and adjusted for using inverse probability weighting (IPW) (Seaman and White 2013).

To fully describe the prevalence of diseases among the private cord blood bank population, three different measures were calculated based on respondent self-report. First, an overall condition prevalence was calculated by dividing the number of unique families that reported at least one of the listed transplant or regenerative indications by the total number of families responding to the survey. Second, a transplant indication prevalence was calculated as the proportion of families reporting at least one first-degree member with a transplant indication potentially treatable with an allogeneic cord blood transplant. Donor children were excluded from this analysis since autologous transplant would be less likely for these indications. Third, a regenerative indication prevalence was calculated as the proportion of families reporting at least one child with a regenerative indication potentially treatable with an autologous cord blood infusion. Summary prevalences are reported with 95 % confidence intervals. The data were analyzed using Stata/IC version 13.0 (StataCorp LP, College Station, TX).

## Results

### Descriptive Analysis

As of May 8, 2015, FHQs were sent to 284,982 eligible families, representing 399,939 children with stored cord blood. Of this group, 94,803 families (33.3 %), representing 134,635 children with stored cord blood units, completed FHQs. Geographically, more than 98 % of the families surveyed (both respondents and non-respondents) resided within the United States. Respondent primary contacts (parents) had an average age of 39.9 years compared to 40.1 for non-respondents. The average number of cord blood units per family were nearly the same in both the respondent and non-respondent groups; however, because of the large sample size, small differences (e.g. between 1.42 and 1.39 cord blood units per family) were still statistically significant. Children with stored units were between 0 and 23 years of age, with the children in the respondent group being slightly younger than the children in the non-respondent group (average age 6.0 vs. 6.4 years old, respectively). To account for these differences, adjusted prevalence rates were calculated using IPW (Electronic Resource 2).

### Family Health Questionnaire Conditions Indicated

Among families with stored UCB, the IPW-adjusted counts showed that 1757 (1.86 %, 95 % CI 1.77–1.95 %) unique families reported one or more of the 16 transplant indications, and 13,706 (14.50 %, 95 % CI 14.27–14.73 %) unique families reported one or more of the 16 regenerative indications. Thus, 16.36 % of families reported at least one transplant indication and/or at least one regenerative indication. The majority of families (83.7 %) reported only one condition and very few (0.35 %) reported both a transplant and a regenerative condition. After accounting for the small number of families that reported both a transplant and a regenerative indication, 16.01 % (95 % CI 15.77–16.25 %) of unique respondent families indicated at least one of the diseases specified on the FHQ.

Table 1 (Transplant indication prevalence) shows the adjusted frequency of transplant indications among families storing cord blood at a private bank. In addition, this table provides a more conservative estimate of the frequency of transplant indications by excluding families with a transplant indication only in a child with stored cord blood, since autologous infusions are less likely for transplant indications in these children. For example, 323 families providing FHQ responses indicated that a member of the immediate family (father, mother, or children, possibly including the child with stored cord blood) had a diagnosis of Non-Hodgkin’s Lymphoma. After excluding families in which the child with the stored cord blood had Non-Hodgkin’s Lymphoma, 314 families remained. With this refinement, 1.64 % (95 % CI 1.55–1.72 %) of unique respondent families had at least one first-degree relative, excluding the donor child, with a transplant indication. The most common transplant indications reported were Non-Hodgkin’s Lymphoma (0.33 %), Hodgkin’s Lymphoma (0.30 %), and Acute Lymphoblastic Leukemia (0.28 %).

**Table 1:** Transplant indication prevalence (N = 94,539 respondent families)

Indication^a^	Families reporting transplant indication (%)^b^	Families reporting transplant indication (not in child donor) (%)^b^
Non-Hodgkin’s lymphoma	323 (0.34)	314 (0.33)
Hodgkin’s lymphoma	290 (0.31)	287 (0.30)
Acute lymphoblastic leukemia	328 (0.35)	263 (0.28)
Sarcoma	144 (0.15)	125 (0.13)
Acute myelogenous leukemia	113 (0.12)	106 (0.11)
Sickle cell disease	121 (0.13)	105 (0.11)
Beta thalassemia major	97 (0.10)	90 (0.10)
Chronic lymphocytic leukemia	88 (0.09)	85 (0.09)
Chronic myelogenous leukemia	82 (0.09)	82 (0.09)
Neuroblastoma	90 (0.10)	61 (0.06)
Multiple myeloma	60 (0.06)	58 (0.06)
Severe aplastic anemia	49 (0.05)	45 (0.05)
Myelodysplastic syndrome	20 (0.02)	19 (0.02)
Diamond–Blackfan anemia	10 (0.01)	9 (0.01)
Fanconi anemia	8 (0.01)	6 (0.01)
Hurler syndrome	0 (0.00)	0 (0.00)
Families reporting at least 1 transplant indication	1823 (1.93)	1655 (1.75)
Unique families reporting at least 1 transplant indication	1757 (1.86)^c^	1546 (1.64)^c^

^a^Families may report more than one indication. Ordered by percent of families reporting indication (not in child donor)

^b^Adjusted using inverse probability weighting based on respondent age, age of child, and number of cord blood units

^c^Percent represents total unique families reporting at least one transplant indication divided by the total families responding. This total is lower than the sum of all specific transplant indications reported because families with more than 1 transplant indication were only counted once here

Table 2 (Regenerative indication prevalence) shows the adjusted frequency of regenerative indications among the responding families. Since regenerative conditions are more likely to be treated with autologous infusions, this table provides a more conservative estimate of the frequency of regenerative indications by only including families with a regenerative indication in a child with stored UCB. For example, 2885 families providing FHQ responses indicated that a member of their immediate family (father, mother, or children) had a diagnosis of Autism/Autism Spectrum Disorder (ASD)/Apraxia. After excluding families in which the member with Autism/ASD/Apraxia was not the child with stored UCB, 1820 families remained. After this refinement, 4.23 % (95 % CI 4.10–4.36 %) of families had at least one child with stored UCB and a regenerative indication. The most common regenerative indications reported were Autism/ASD/Apraxia (1.93 %), Other Developmental Delay (1.36 %), and Congenital Heart Defect (0.87 %).

**Table 2:** Regenerative indication prevalence (N = 94,539 families)

Indication^a^	Families reporting regenerative indication (%)^b^	Families reporting regenerative indication (only in child donor) (%)^b^
Autism/ASD/apraxia	2885 (3.05)	1820 (1.93)
Other developmental delay	2119 (2.24)	1282 (1.36)
Congenital heart defect^c^	230 (1.86)	107 (0.87)
Childhood hearing loss	1097 (1.16)	378 (0.40)
Diabetes, type I	2374 (2.51)	247 (0.26)
Cerebral palsy/PVL/hypotonia	748 (0.79)	234 (0.25)
Inflammatory bowel disease	2218 (2.35)	128 (0.14)
Hydrocephalus	278 (0.29)	121 (0.13)
In-utero brain injury/stroke	233 (0.25)	98 (0.10)
Hypoxic-ischemic brain injury	270 (0.29)	77 (0.08)
Traumatic brain injury	344 (0.36)	54 (0.06)
Infant lung disease (e.g. bronchopulmonary dysplasia)	100 (0.11)	49 (0.05)
Spinal cord injury	348 (0.37)	33 (0.03)
Muscular dystrophy	156 (0.17)	33 (0.03)
Diabetes, type II	2592 (2.74)	12 (0.01)
Systemic lupus	431 (0.46)	4 (0.00)
Families reporting at least 1 regenerative indication	16,423 (17.37)^d^	4677 (4.95)^d^
Unique families reporting at least 1 regenerative indication	13,706 (14.50)^e^	4000 (4.23)^e^

*ASD* autism spectrum disorder, *PVL* periventricular leukomalacia

^a^Families may report more than one indication. Ordered by percent of families reporting indication (only in child donor)

^b^Adjusted using inverse probability weighting based on respondent age, age of child, and number of cord blood units

^c^Congenital heart defect appeared only on a subset of the surveys of which 12,366 were returned. Therefore, we calculated the prevalence based on this lower denominator

^d^The sum of the percentages for all specific indications is more than the total number of regenerative indications divided by the sampled population because the prevalence for congenital heart defect was calculated on a subset of families

^e^Percent represents unique families reporting at least one regenerative indication divided by total unique families responding. The total is lower than the sum of regenerative indications (families with more than 1 regenerative indication were counted once)

Although IPW-adjusted transplant, regenerative, and specific indication proportions are reported, weighted and unweighted figures did not differ materially (Electronic Resources 3 and 4).

## Discussion

While stem cell treatment is still a developing field, the identification of UCB as a relatively abundant and ethically uncontroversial source of stem cells has made cord blood banking an important topic (Forraz and McGuckin 2011). To our knowledge, this is the first cross-sectional study of disease prevalence among families with stored UCB at a private cord blood bank. From a list of 32 conditions, our survey of families who have banked UCB estimated that 16.01 % of unique families reported at least one disease or condition currently treated with, or under investigation for treatment with, UCB. Furthermore, after excluding autologous cord blood infusions for traditional transplant indications and allogeneic transplants for regenerative indications, 1.64 % of unique families had at least one first-degree member with a transplant condition potentially amenable to allogeneic cord blood transplant while 4.23 % of unique families had at least one child with a regenerative condition potentially amenable to receiving an autologous cord blood infusion.

Although this may be the first study to publish the prevalence of various conditions in families with privately stored UCB, other studies have looked at the likelihood of utilization of banked UCB. For example, in 1997, Johnson estimated the probability of clinical need for UCB by multiplying the probability of developing a disease (based on prevalence in the general population), by the probability of need for transplantation (vs. other first-line therapies) (Johnson 1997; Ballen et al. 2008). As part of his analysis, Johnson estimated that 608 (0.30 %) of 200,000 babies born each year face the risk of developing cancer or another life threatening hematopoietic, immunodeficiency, or genetic disease before adulthood. However, Johnson went on to suggest that fewer than half of these children (216, or 0.11 %) could actually benefit from an autologous or allogeneic stem cell transplantation of UCB. Another estimate of the likelihood of stem cell transplant was performed more recently (Nietfeld et al. 2008). Nietfeld et al. estimated the lifetime probability of hematopoietic stem cell transplantation in the United States under four distinct scenarios using data from the Center for International Blood and Marrow Transplant Research, the United States Surveillance, Epidemiology and End Results Program, and the United States Census Bureau. It was estimated that the cumulative lifetime probability of undergoing a hematopoietic stem cell transplant ranged from 0.23 to 0.98 %.

This study’s prevalence estimates do not measure the need for transplant; rather they measure the prevalence of conditions that might be amenable to treatment with transplant or infusion in a subset of those individuals. In fact, there are many reasons why a patient or family member with stored UCB might not receive a transplant or infusion despite being diagnosed with a condition that is potentially treatable with stem cells. For example, many of the conditions amenable to UCB transplant are malignancies, like lymphoma, with established treatments such as radiation or chemotherapy that are often used successfully prior to stem cell transplant (Isidori et al. 2015). For allogeneic transplants, there may not be an appropriate HLA match since, for example, only 1 in 4 siblings of the same biological parents are a haplo-identical HLA match (Butler and Menitove 2011). Furthermore, some UCB units may contain insufficient cell dose to be successfully used for transplant, particularly in older children and adults (Page et al. 2011). Other barriers to UCB infusion or transplant may exist, including educational, cultural, and socioeconomic factors. Longitudinal studies of this population may help to better characterize and understand these barriers.

There are other substantive differences between the prevalence estimates in this study and the likelihood of transplant estimates from previous reports. Of note, neither Johnson nor Nietfield accounted for the allogeneic transplantation of UCB units to family members other than the child with stored cells. Furthermore, neither study incorporated the potential use of UCB units in patients with regenerative conditions.

This study has several limitations. The use of self-report without clinical confirmation may have introduced misclassification of individuals and families. Also, our response rate was 33.3 %, raising the question of non-response bias. To help address this concern, we adjusted our results using IPW. Furthermore, since study families paid to store cord blood privately, they likely represent a group with higher average socioeconomic status than the general population, which could reduce the generalizability of this study’s finding to populations beyond private cord blood banks. However, since the goal of this study was to characterize the population of families storing cells in private cord blood banks, generalizability beyond this group was not a key objective.

Millions of units of UCB are stored in private cord blood banks. Before this study, the medical community had little understanding of the conditions faced by the families who choose to store their children’s UCB. This prevalence data, combined with the results of ongoing clinical trials to document the clinical impact of UCB for new indications, should lead to a better understanding of the true potential of this unique resource.

## Electronic Supplementary Material

Below is the link to the electronic supplementary material.

Supplementary material 1 (DOCX 22 kb)
