# Supplementary material for: Prevalence of Medical Conditions Potentially Amenable to Cellular Therapy among Families Privately Storing Umbilical Cord Blood
Source: Matern Child Health J. 2016 Aug 16;21(1):208–14. doi: 10.1007/s10995-016-2110-1 (PMC5226972; doi:10.1007/s10995-016-2110-1)
Supplement: Supplementary file 1 — Supplementary material 1 (DOCX 22 kb) [file 10995_2016_2110_MOESM1_ESM.docx]

**Electronic Supplemental Materials**

**Electronic Resource 1**

US Based Clinical Trials Studying Infusion of Umbilical Cord Blood in Regenerative Applications in Pediatric Populations (as of August 2015)

| **Condition** | **Clinical Trials ID/Link** | **Institution** |
| --- | --- | --- |
| Autism | NCT01638819 | Sutter Health |
|  | NCT02176317 | Duke University |
| Cerebral Palsy | NCT01072370 | Georgia Regents University |
|  | NCT01988584 | The University of Texas Health Science Center, Houston |
|  | NCT01147653 | Duke University |
| Hearing Loss | NCT02038972 | Florida Hospital |
| Hypoplastic Left Heart Syndrome | NCT0144504 | Duke University |
|  | NCT01883076 | Mayo Clinic |
| Hypoxic-Ischemic Encephalopathy | NCT00593242 | Duke University |
| Pediatric Stroke | NCT02460484 | Florida Hospital |

**Electronic Resource 2**

Inverse Probability Weighting of Demographic Variables (N=94,539 families)

|  | **Mean in entire surveyed population** | **Mean among respondents** | **Weighted mean among respondents** |
| --- | --- | --- | --- |
| Primary account holder’s age | 40.0 | 39.9 | 40.1 |
| Average age of children | 6.3 | 6.0 | 6.3 |
| Number of stored units | 1.4 | 1.4 | 1.4 |

To investigate the potential impact of non-response bias, a model was created to adjust for non-response based on available demographic characteristics using inverse probability weighting. This methodology assigns weights to each respondent to rebalance the set of respondents so that it becomes representative of the whole sample.

**Electronic Resource 3**

Inverse Probability Weighting of Transplant Indications (N=94,539 families)

| **Indication** | **Observed families reporting transplant indication (not in child donor)** |  | **Weighted number of families reporting transplant indication (not in child donor)** ^a^ |
| --- | --- | --- | --- |
| Non-Hodgkin's Lymphoma | 305 |  | 314 |
| Hodgkin's Lymphoma | 284 |  | 287 |
| Acute Lymphoblastic Leukemia | 261 |  | 263 |
| Sarcoma | 123 |  | 125 |
| Acute Myelogenous Leukemia | 104 |  | 106 |
| Sickle Cell Disease | 108 |  | 105 |
| Beta Thalassemia Major | 91 |  | 90 |
| Chronic Lymphocytic Leukemia | 83 |  | 85 |
| Chronic Myelogenous Leukemia | 82 |  | 82 |
| Neuroblastoma | 60 |  | 61 |
| Multiple Myeloma | 57 |  | 58 |
| Severe Aplastic Anemia | 43 |  | 45 |
| Myelodysplastic Syndrome | 20 |  | 19 |
| Diamond-Blackfan Anemia | 11 |  | 9 |
| Fanconi Anemia | 6 |  | 6 |
| Hurler syndrome | 0 |  | 0 |
| **Unique families reporting at least 1 transplant indication** | **1,532 (1.62%)** |  | **1,546 (1.64%)** |

^a^ Each family’s weight is the inverse of their probability of being a respondent (using primary account holder’s age, average age of children, and number of stored units as predictor variables) multiplied by the overall response rate.

**Electronic Resource 4**

Inverse Probability Weighting of Regenerative Indications (N=94,539 families)

| **Indication** | | **Observed families reporting regenerative indication (only in child donor)** | |  | | **Weighted number of families reporting regenerative indication (only in child donor)** ^a^ |
| --- | --- | --- | --- | --- | --- | --- |
| Autism/ASD/Apraxia | | 1,763 | |  | | 1,820 |
| Other Developmental Delay | | 1,265 | |  | | 1,282 |
| Congenital Heart Defect | | 111 | |  | | 107 |
| Childhood Hearing Loss | | 367 | |  | | 378 |
| Diabetes, Type I | | 226 | |  | | 247 |
| Cerebral Palsy/PVL/Hypotonia | | 236 | |  | | 234 |
| Inflammatory Bowel Disease | | 117 | |  | | 128 |
| Hydrocephalus | | 126 | |  | | 121 |
| In-utero Brain Injury/Stroke | | 100 | |  | | 98 |
| Hypoxic-ischemic Brain Injury | | 78 | |  | | 77 |
| Traumatic Brain Injury | 51 | | |  | 54 | |
| Infant Lung Disease (e.g., Bronchopulmonary Dysplasia) | | | 51 |  | | 49 |
| Spinal Cord Injury | | 33 | |  | | 33 |
| Muscular Dystrophy | | 33 | |  | | 33 |
| Diabetes , Type II | | 11 | |  | | 12 |
| Systemic Lupus | | 4 | |  | | 4 |
| **Unique families reporting at least 1 regenerative indication** | | **3,989 (4.21%)** | |  | | **4,000 (4.23%)** |

^a^ Each family’s weight is the inverse of their probability of being a respondent (using primary account holder’s age, average age of children, and number of stored units as predictor variables) multiplied by the overall response rate.
